# A Computational Model of Peripheral Photocoagulation for the Prevention of Progressive Diabetic Capillary Occlusion

**DOI:** 10.1155/2016/2508381

**Published:** 2016-10-25

**Authors:** Thomas J. Gast, Xiao Fu, John Scott Gens, James A. Glazier

**Affiliations:** ^1^School of Optometry, Indiana University, Bloomington, IN 47405, USA; ^2^The Biocomplexity Institute, Indiana University, Bloomington, IN 47405, USA; ^3^Physics Department, Indiana University, Bloomington, IN 47405, USA; ^4^School of Informatics and Computing, Indiana University, Bloomington, IN 47408, USA

## Abstract

We developed a computational model of the propagation of retinal ischemia in diabetic retinopathy and analyzed the consequences of various patterns and sizes of burns in peripheral retinal photocoagulation. The model addresses retinal ischemia as a phenomenon of adverse local feedback in which once a capillary is occluded there is an elevated probability of occlusion of adjacent capillaries resulting in enlarging areas of retinal ischemia as is commonly seen clinically. Retinal burns of different sizes and patterns, treated as local oxygen sources, are predicted to have different effects on the propagation of retinal ischemia. The patterns of retinal burns are optimized with regard to minimization of the sum of the photocoagulated retina and computer predicted ischemic retina. Our simulations show that certain patterns of retinal burns are effective at preventing the spatial spread of ischemia by creating oxygenated boundaries across which the ischemia does not propagate. This model makes no statement about current PRP treatment of avascular peripheral retina and notes that the usual spot sizes used in PRP will not prevent ischemic propagation in still vascularized retinal areas. The model seems to show that a properly patterned laser treatment of still vascularized peripheral retina may be able to prevent or at least constrain the propagation of diabetic retinal ischemia in those retinal areas with intact capillaries.

## 1. Introduction

Panretinal photocoagulation (PRP) has had a long history of success in the treatment of neovascularization seen in diabetic retinopathy. Supported by the Diabetic Retinopathy Study [1] and the Early Treatment Diabetic Retinopathy Study [2] PRP has successfully treated diabetic neovascularization with the treatment pattern consisting of 1200–1600 spots of 250–500 microns in size, delivered to the peripheral retinal areas usually over 2 treatment sessions [3]. This has remained stable until the last few years in which certain modifications have been introduced by use of the multispot pattern scan laser (PASCAL) [4, 5] generally using a rectangular grid pattern of retinal burns of short temporal duration and also targeted PRP [6, 7] which treats only ischemic retina as determined by wide field angiography. The detailed physiology of photocoagulation is in some debate but the most common interpretation is that photocoagulation destroys photoreceptors which act as oxygen sinks in areas of retinal ischemia. This destruction removes a consumer of oxygen and introduces oxygen from the choroidal vasculature, yielding areas of increased oxygenation, reducing the ischemic retinal area, and lowering vascular endothelial growth factor (VEGF) production by ischemic retina. This lowering of VEGF production then results in involution of the neovascularization which motivated treatment. This paper introduces another rationale for panretinal photocoagulation with possible implications for therapy. Our previous modelling work [8] was on the progression of retinal ischemia due to an adverse local feedback cycle in which occlusion of a single retinal capillary increases the likelihood of occlusion of adjacent capillaries. This model was developed to explain the apparent inconsistency between a mechanism of individual capillary occlusion based on leukocyte adhesion and the large areas of contiguous capillary loss seen clinically as the dark areas on wide field fluorescein angiography. Although we have chosen not to model the precise mechanism by which the capillary occlusion propagates in full molecular detail, current literature suggests that some of the molecules responsible may indeed be known. In the presence of elevated glucose, leukocytes are known to express increased levels of ICAM receptors [9–11]. ICAM expression on the surface of endothelial cells has been reported to be elevated in response to increases in VEGF [12], which would allow leukocytes to adhere to and trigger a localized occlusion event. These two alterations in the physiology of diabetes act as core mechanisms in the assumptions driving the model in discriminating the diabetic from the normal. While there is much physiological support for the noncanonical role of VEGF as a central factor in capillary occlusion acting through an elevation of endothelial ICAMs [8], in our model VEGF is regarded as a surrogate for the complex balance of it and various additional cytokines such as angiopoietin-2 and pigment epithelial derived factor. In our previous modelling study [8], we focused on treating diffusing VEGF as the important spatially propagating signal, which linked local capillary closure-induced retinal hypoxia with derived occlusion of adjacent capillaries. We ignored the mechanistic complexity of leukostasis involving various molecular factors and used occlusion probability function dependent on local blood flow velocity and VEGF level to describe the likelihood of emergence of capillary occlusion. In our prior simulations, areas of retinal ischemia tended to propagate, increasing in size over time, but to be restrained by the oxygenated areas surrounding larger retinal vessels. This observation provided the motivation for a computational model which we use in this paper to analyze photocoagulation as a treatment creating oxygenated areas, particularly with regard to prevention of progressive retinal ischemia. Clinically, current peripheral photocoagulation is done in the context of retinal neovascularization. It is done to the entire peripheral retina; that is, regardless of whether a given retinal area is ischemic or not, that area is photocoagulated. This approach is that utilized in the Diabetic Retinopathy Study and partly it is mandated by necessity in that wide field angiography is required to discriminate vascularized from ischemic peripheral retina. The exceptions to this approach are those relatively few ophthalmologists who routinely use a targeted retinal photocoagulation approach treating only ischemic retinal areas after their identification by wide field angiography [6, 7].

Within the context of our model, we have analyzed whether particular patterns of retinal photocoagulation can possibly prevent progressive capillary loss in areas of peripheral retina not yet ischemic. It is rational and important to treat patients with diabetes in a way which can prevent progressive loss of capillaries in those areas not already ischemic since retinal ischemia is a major driver of neovascularization through the elevation of VEGF [13–15]. This approach would only be beneficial in the treatment of retinal areas which still possess intact capillaries. Our present model allows the testing of different patterns and sizes of burns and results in the prediction that progression of diabetic retinal ischemia can be greatly reduced by using a pattern of small laser burns quite different to that utilized in standard PRP.

## 2. Materials and Methods

We start by constructing a conceptual model with physiologically based model assumptions. We assume that Mueller cells are the sole retinal source of VEGF in diabetic retina [16–18] and assume a slight elevation of VEGF production by Mueller cells in a diabetic retina above that in the normal retina [19, 20]. This is the permissive step distinguishing diabetics with slightly elevated endothelial ICAMs induced by the slightly elevated VEGF from normal cases. This assumption allows us to computationally treat retinal tissue as two cell groups: Mueller cells and other retinal cells. We further assume that the vascular supply to each area of retina is “critical” in that occlusion of a capillary will result in ischemia of an area of physiologically dependent retina with a resultant considerable further local elevation of VEGF synthesis [21] by hypoxic Mueller cells above the already somewhat elevated permissive level of VEGF [22–24]. This assumption guarantees that in the model initial capillary occlusion would always lead to some hypoxia of retinal tissue, which is a necessary condition for differentiation of untreated and treated simulations.

Our key point is that retinal ischemia propagates as a phenomenon of adverse local feedback in which once a capillary is occluded there is an elevated probability of occlusion of adjacent capillaries. We make the following assumptions relevant to this adverse local feedback which are essential to development of our model. As discussed above, we treat VEGF as a surrogate of the collective actions of it and other cytokines. We assume that VEGF functions as the mediator of the said adverse local feedback. VEGF is produced by individual Mueller cells in variable amounts based on local cellular level oxygen saturation [21]. VEGF secreted by Mueller cells diffuses and is consumed by the various cells including capillary endothelial cells. Capillary occlusion is assumed to be probabilistic, functionally based on local VEGF level and the calculated blood flow velocity in the capillary segments. The higher the VEGF concentration and the smaller the blood flow velocity, the greater the chance a capillary becomes occluded. We further assume that capillary occlusion is irreversible. This assumption allows us to remove a blood flow pathway permanently from the vascular network once occlusion occurs. As an extension to model assumptions in prior study [8], we further assume in the present study that photocoagulation burns are effectively diffusive sources of oxygen of a size and shape determined by a coagulation burn. We predict that photocoagulation burns of proper patterns and sizes, as oxygen sources, would oxygenate otherwise hypoxic Mueller cells, lowering their local synthesis of VEGF and thereby reduce the probability of adjacent capillary occlusion as this probability is dependent on local VEGF concentration.

**Figure 1:**
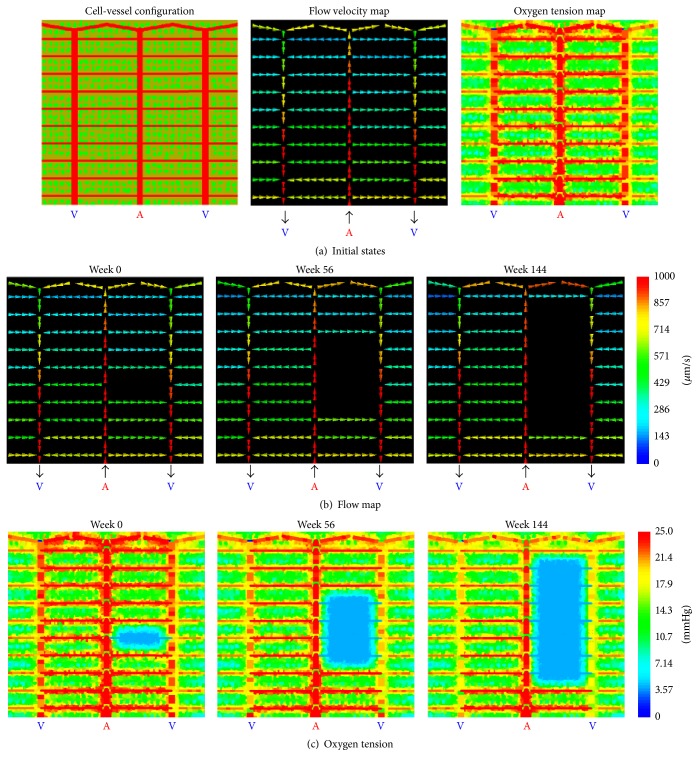
Pattern of capillary loss without burns. Normal condition of peripheral retina without photocoagulation. (a) (left) A 2D view of the configuration of the retinal capillary network, Mueller cells and other retinal cells in the* in silico* peripheral retina. Size of a Mueller cell (green) is 24 *μ*m [39]. Size of other retinal cells (olive) is 21 *μ*m. Diameter of arteriole (red) is 20 *μ*m. Diameter of venules (red) is 25 *μ*m. Diameter of shunting vessels at the top of the model area (red) is 18 *μ*m. Capillary (red) diameter is 10 *μ*m. Vessel pattern and diameters are from [25]. (Middle) flow velocity map of the vascular network. Capillaries carry blood flow with smaller velocities than arterioles, venules, and shunting vessels. (Right) oxygen tension map of the modelled section. Cells in the proximity of vessels have relatively higher oxygenation and redder colors than those located at a greater distance but no area is ischemic in the normal condition. (b) Evolution of the flow velocity map following initial capillary occlusion without photocoagulation. (Left) flow map shows closure of a capillary in AV sector. (Middle and right) a cascade of capillary occlusion instances propagates anteriorly and posteriorly within the same sector, and therefore the flow map shows a progressively larger gap. Color bar has units *μ*m/s. Warmer color represents greater flow velocity. (c) Evolution of the oxygen tension map following initial capillary occlusion without photocoagulation. (Left) oxygen tension map shows a group of poorly oxygenated cells near occluded vessel. (Middle and right) area of the ischemic region expands in response to the increasing number of derived capillary occlusion instances. Also oxygen tension in the venules drops with time. Color bar has unit mmHg. Warmer color represents higher oxygen tension.

**Figure 2:**
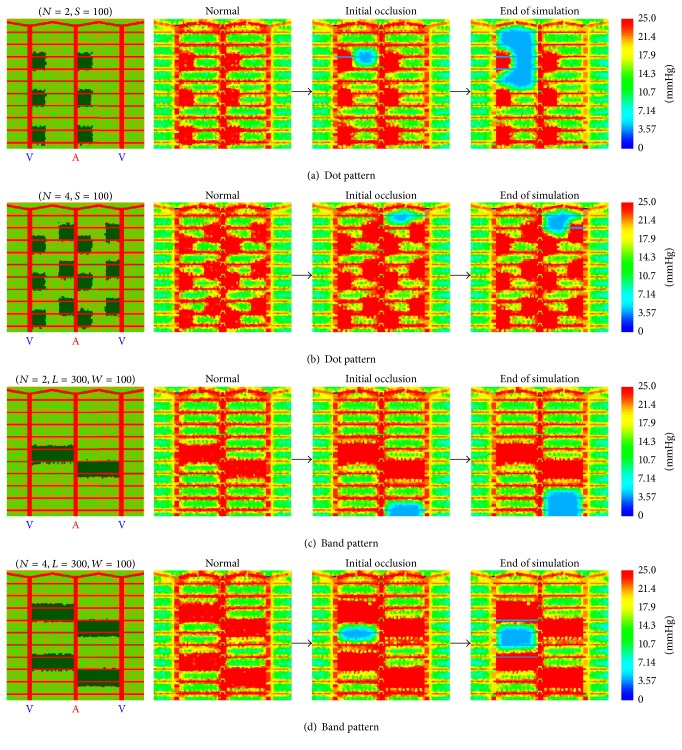
Dot and band burns patterns: effects of density. Effects of dot and band laser burn density on ischemia progression.* Dot pattern* burn (first subfigure rows (a) and (b)) with *N* = 2 (a) and *N* = 4 (b) and their effectiveness on prevention of ischemia progression (second to fourth subfigures in each row). Label *N* = 2 indicates that the initial sum ablated area of these* dot pattern* burns is equivalent to that with two* band pattern* burns. Label *S* = 100 means that the edge of the* dot pattern* burn is 100 microns.* Band pattern* burns (first subfigure rows (c) and (d)) with *N* = 2 (c) and *N* = 4 (d) and their effectiveness on prevention of ischemia progression (second to fourth subfigures in each row). Label *N* = 2 means that number of laser burns is 2. Label “*L* = 300, *W* = 100” means that* band pattern* burn has length of 300 microns and width of 100 microns. Color bar for oxygen maps has unit mmHg. Warmer color in oxygen maps represents higher oxygen tension.

We translate the conceptual model into a computational model with the introduction of the following model components and processes. Our model is composed of four primary components: Mueller cells (green in “cell-vessel configuration” in Figure 1(a)), other retinal cells (olive in “cell-vessel configuration” in Figure 1(a)), capillary blocks (red in “cell-vessel configuration” in Figure 1(a)), and photocoagulated retinal cells (dark green in “dot pattern” in Figure 2(a)). We then construct a retinal vasculature composed of capillary blocks based on a schematic peripheral retinal capillary network based on the histology of Spitznas and Bornfeld [25] and uniformly pattern Mueller cells and other retinal cells into the space between blood vessels in the control group. In the experimental groups, specific patterns of laser burns are composed of photocoagulated retinal cells.

Next, we apply a hemodynamic model [8] to calculate blood flow velocities across the whole vascular network (“flow velocity map in Figure 1(a)”). We then mathematically model oxygen advection within the vasculature, oxygen diffusion between vessels and cells, and oxygen consumption by Mueller cells and other retinal cells. Once the oxygen steady state is established, we model VEGF synthesis in Mueller cells, VEGF diffusion between cells and vessels, and VEGF decay in cells. At time zero, the model introduces a random occlusion of a capillary and then calculates a reestablished steady state of oxygen and VEGF. The rest of the simulation periodically examines the occlusion probability function for individual capillaries. If a new occlusion occurs, the model removes the relevant blood flow path and repeats the calculation of blood flow velocities and simulation of the oxygen and VEGF steady states [8]. We implement the computational model in CompuCell3D software [26].

To present simulation results, we show capillary network structure, blood flow, and oxygen tensions for selected simulation runs. Additionally, using box plots, we summarize simulated efficacy of laser therapy based on retinal area photocoagulated and calculated retinal ischemia from replicate simulations. Shown in Figure 1(a) is the configuration of retinal periphery cells and vessels under normal diabetic conditions, visualized at a two-dimensional plane involving vasculature. With boundary blood pressures and oxygen tensions assigned, flow velocities were calculated and the oxygen steady state was simulated (Figure 1(a)). Larger vessels including the arteriole, venules, and the peripheral shunt vessels showed greater blood flow velocities than do the capillaries. All cells were well-oxygenated under normal conditions, and cells adjacent to vessels had higher oxygen tension than those distant from vessels. Figures 1(b) and 1(c) show the progression of capillary occlusion in terms of the flow map and the oxygen tension map with the occlusion occurring at week 0 and results shown in model weeks 56 and 144. Into the peripheral retinal capillary networks distinct patterns of laser burns were applied and the area of retina treated and the predicted area of retinal ischemia over the time were modelled. The sum of these two areas acts as a measure of optimization for a particular pattern of retinal photocoagulation (see Section 4).

## 3. Results 

### 3.1. Peripheral Retinal Capillary Network Shows Little Resistance to Progression of Capillary Occlusion without Laser Treatment

Our simulations showed that an initial capillary occlusion, without laser treatment, often but not always, led to a cascade of derived occlusion instances and large contiguous ischemic areas confined by an arteriole and venule (Figures 1(b) and 1(c)). A stochastic occurrence of capillary occlusion broke a capillary flow pathway and led to an ischemic region (Figure 1(b) left) and resulting local hypoxia (Figure 1(c) left). Hypoxic Mueller cells significantly elevated local synthesis of VEGF which diffused to other nearby vessels, raising the probability of their occlusion (not shown in figure but detailed in [8]). The time point of this initial capillary occlusion was denoted as week 0. Thanks to their comparably large flow velocity and large luminal diameters, the arteriole and venules are relatively protected from occlusion as they are in the clinical situation until late in the ischemic process when flows drop. Derived capillary occlusion emerged at other nearby capillaries. Within the model this gives an anterior-posterior expansion of the no-flow region (Figure 1(b), middle and right) and of the corresponding retinal hypoxia (Figure 1(c)).

### 3.2. Dotted and Banded Photocoagulation Patterns Utilizing Small Burns Can Effectively Prevent Progression of Capillary Occlusion

The underlying mechanism of photocoagulation is the ablation of photoreceptors, which are sinks of oxygen delivered from choroidal capillaries, so as to create an additional oxygen source for the inner retina. Common panretinal photocoagulation uses laser burns with a typical size of 250–500 microns [2]. Our model indicates that regularly patterned laser burns of much smaller sizes, likely causing less damage to retinal tissue, would have the potential to effectively prevent progression of capillary occlusion in diabetic retinopathy. For the sake of clear illustrations, we chose to only use normal, beginning, and eventual (3 model years) oxygen tension maps as descriptors of progression of capillary occlusion in the following case studies of simulated laser therapies. For simplicity we only present the oxygen maps and not flow or VEGF maps.

We initially tested two basic patterns of laser photocoagulation burns with the same total ablated area of retinal tissue (Figure 2). One pattern considered was square dotted laser burns alongside the arteriole and venule, each burn with size of 100 microns by 100 microns. The other pattern tested was rectangular banded laser burns connecting the arteriole and venule in a perpendicular orientation, each with size of 300 microns (the arteriolar/venular distance) by 100 microns. All burns were shown as dark patches in the figures. Various densities of such basic laser burns were considered labelled with “*N*” followed by a number. For example, *N* = 2 referred to 2 bands of laser burns placed in the modelled region in* band pattern* simulations, and *N* = 2 also stood for the number of dotted laser burns in* dot pattern* simulations that would give the same total burn area as 2 banded burns did in the* band pattern* simulations. We clarify that the sizes of burns given in the* dot pattern* or* band pattern* were mathematically intended values. In practice, burn sizes were normally not precisely equivalent to but always close to these values in different simulations. In the model photocoagulated tissue is cells which have lost their physiological functions such as consumption of oxygen. A mathematically square burn has varying overlap with cells at its edges the degree of which determines the life or death of a cell and thus gives slight imprecision in burn size. This is seen in Figure 2 if the burn edges are carefully examined. Also note that we limited our treatment to the middle two simulated intact arteriole-venule sectors, by placing laser burns in only these two sectors and by limiting candidate sites of random initial occlusion to capillaries within these two sectors. We believe that similar laser patterns and the simulated results can be applied for larger spatial scales composed of more sectors.

Figure 2 shows four case studies of photocoagulation therapies using* dot pattern* (Figures 2(a) and 2(b)) and* band pattern* (Figures 2(c) and 2(d)) laser burns. As the figure illustrates, ablated areas were enriched in oxygen under the normal condition and throughout the entire simulation secondary to oxygen delivery from the choroidal vasculature. In the* dot pattern* case with *N* = 4, an initial capillary occlusion stochastically occurred adjacent to the shunting vessel and at the end of simulation progressed to cause derived capillary occlusion (Figure 2(b)). Apparently, the photocoagulated regions supplied adequate oxygen, preventing hypoxia, and elevated local VEGF synthesis, so the occlusion of the second capillary failed to progress to a larger ischemic area. Furthermore, the location of the ischemic region was close to well-oxygenated regions with low VEGF, making it impossible to propagate to other intact retinal areas. In contrast, the dot pattern with *N* = 2 was incapable of preventing progression of ischemia, evidenced by the “bypassing” pattern of hypoxia at the end of simulation though there was still less progression of capillary occlusion than that seen in the untreated case. In the* band pattern* with *N* = 4, random initial occlusion emerged in the middle zone of a sector and consequently stimulated additional capillary occlusion but at the end of simulation ischemia remained spatially confined (Figure 2(d) right) with ischemic propagation unable to cross either a photocoagulation band or an arteriole or venule.* Band pattern* with *N* = 2 showed a very similar preventive effect on ischemic progression but areas of possible ischemia are larger. Similar to the* dot pattern* case with *N* = 4, the region of photocoagulation acted as an oxygen source implying low VEGF and created a barrier to propagation of capillary occlusion. To briefly sum up, both simulated *N* = 4* band pattern* and* dot pattern* photocoagulation effectively inhibit progression of diabetic capillary occlusion. Similar preventive functions were also found in *N* = 6* band pattern* and* dot pattern* (data not shown), but they have more retinal photocoagulation than is necessary to prevent propagation. In comparison, *N* = 2* dot pattern* therapies did not prevent progression of capillary occlusion and allowed large areas of retinal ischemia, while *N* = 2* Band Pattern*, despite creating effective barriers, allowed significant ischemic progression. Therefore, we selected *N* = 4* band pattern* and* dot pattern*, which seemed near ideal, and further evaluated their efficacy in prevention of ischemic propagation as a function of burn size.

**Figure 3:**
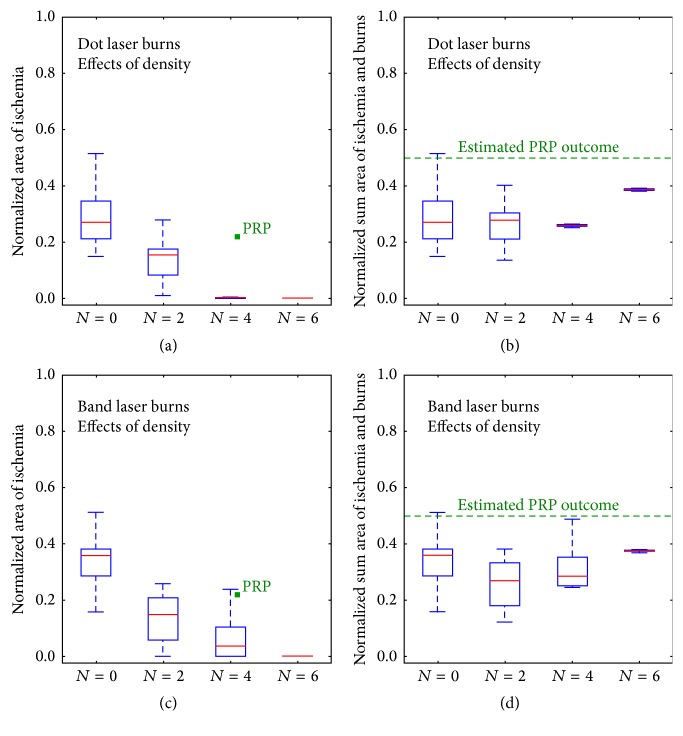
Evaluation of dot pattern and band pattern in terms of coagulated retinal area and predicted ischemic area. Replicate simulations were executed for* dot* and* band patterns*, divided into four *N* groups with *N* = 0, 2, 4, and 6. Note that *N* = 0 represents the control group without photocoagulation whereas other *N*'s indicate the total number of bands and therefor increasing areas of photocoagulation. For a given *N*, the normalized burn areas are essentially equal for the dot and band patterns. *N* is plotted on the *x*-axis, while the fraction of retina which is ischemic, the normalized area at the end of three simulated years, is plotted on the *y*-axis for the* dot* (a) and* band* (c)* patterns*. Each of these panels also includes standard PRP as a green square. Outcomes from replicate simulations are given as box and whisker plots. (b) and (d) plot *N* equals number of bands or of dot burns of equal total area on the *x*-axis and plot the normalized sum of treated and final ischemic areas on the *y*-axis. Estimated PRP outcome is given based on a 0.28 normalized burn area for the total peripheral retinal [2] plus an estimated ischemic propagation for nontreated retina based on the control case.

In order to evaluate efficacy of simulated photocoagulation, we regarded end-of-simulation ischemic area as an important indicator of severity of diabetic progression. Nevertheless, we think of visual function deficit as a consequence of both ischemic area and area of laser ablation as justified in the discussion. With this in mind, we evaluated the sum of the area of initial laser ablation and the area of ischemic propagation as an important metric. We basically define the optimal photocoagulation to have the lowest sum of ischemic area produced by capillary occlusion and tissue damage from photocoagulation. For each of the* dot patterns* and* band patterns* above, we ran replicate simulations to average out the random effects occurring from the stochastic nature of capillary occlusion in a single simulation and then plotted a box plot summarizing these simulation photocoagulation therapies (Figure 3). The control group without any treatment was plotted as 0 area of burn or *N* = 0. In replicate simulations of the same therapy, individual outcomes showed slightly different initial burned areas, which reflected our discussion earlier that intended square or rectangular burned regions would have anatomically fuzzy edges in practice. In Figure 3(a) there are four box and whisker plots for the* dot pattern* of propagated ischemic area versus area of burns for *N* = 0, 2, 4, and 6 patterns, respectively. A strong trend is that the larger the initial burned area, the smaller the ultimate ischemic area. Also shown is that if dot patterns have a total burn area as a fraction of total retina near or larger than *N* = 4 (0.26 normalized retinal area), no apparent progression of ischemia occurs.  Figure 3(b) plots the normalized sum of burn area and ischemia against these different dot treatment conditions and shows little dependence of the sum on area treated with the dot pattern as decreased propagation occurs as normalized burn area is increased though there is an inflection at *N* = 4 showing that laser treatments in excess of *N* = 4 yield greater than necessary total retinal damage. The picture seen in Figures 3(c) and 3(d) for the band pattern is similar to decreased ischemic propagation as burn area increases (Figure 3(c)) but that advantage is being largely cancelled by the greater burn area essentially equaling the decreased ischemic area (Figure 3(d)). A remarkable feature of both dot and band pattern plots is that ischemic propagation can be stopped at normalized burn areas comparable to standard PRP which does not stop ischemic progression because the wide retinal areas between the large burns of standard PRP will behave as the modelled untreated case with ischemic propagation. Therefore, we investigated optimization of laser burn dimensions based on *N* = 4 burn patterns which were the smallest burn area to yield cessation of propagation.

### 3.3. Optimization of Dotted and Banded Laser Patterns

**Figure 4:**
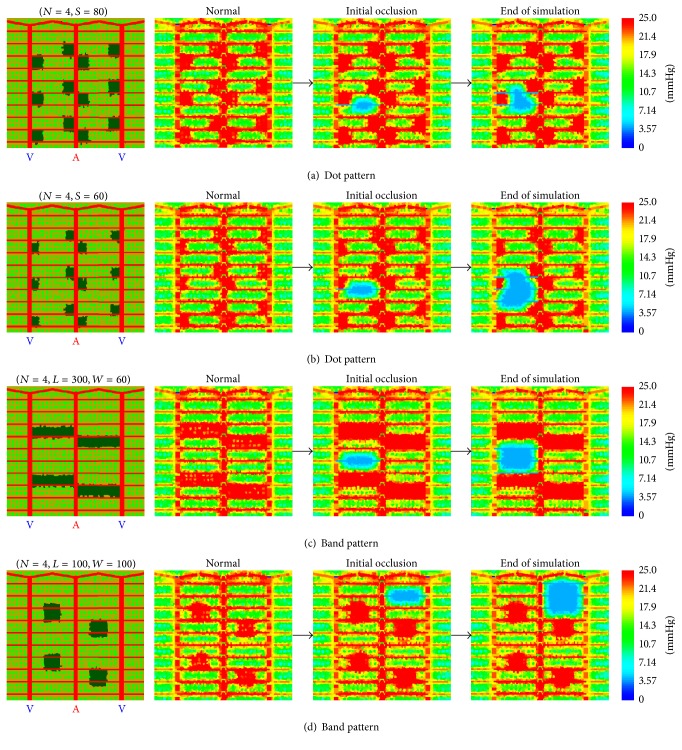
Dot and band burn patterns: effects of size. Size effects of dot and band pattern laser burns on ischemic progression.* Dot pattern* burns (first subfigure rows (a) and (b)) with *S* = 80 microns (a) and *S* = 60 microns (b) and their effectiveness on prevention of ischemic progression (second to fourth subfigures rows (a) and (b)). Label *N* = 4 indicates that the initial total photocoagulated area of these* dot pattern* burns is equivalent to that with *N* = 4* band pattern* burns.* Dot pattern* burn is 40 microns.* Band pattern* burns (first subfigure rows (c) and (d)) with “*L* = 300 microns, *W* = 60 microns” (c) and “*L* = 100 microns, *W* = 100 microns” (d) and their effectiveness on prevention of ischemic progression (second to fourth subfigures rows (c) and (d)). Label *N* = 4 means that the number of laser burns is 4. Color bar for oxygen maps has unit mmHg. Warmer color in oxygen maps represents higher oxygen tension.

**Figure 5:**
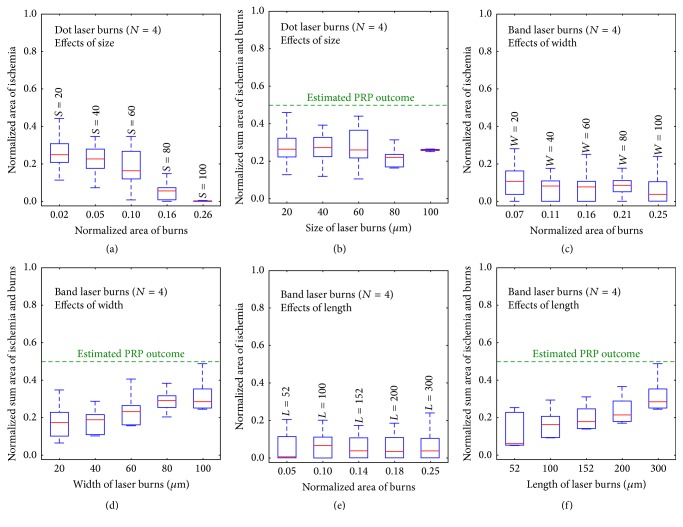
Evaluation of dot pattern size and thinned and shortened band patterns. Replicate simulations with results as box and whisker plots for dot and band pattern burns of different dimensions. Estimated outcome from commonly used panretinal photocoagulation with 1500 burns and 500 micron spots was marked with a green line in panels (b), (d), and (f). (a) Normalized area of dot burns plotted on the *x*-axis and the replicate results plotted on the *y*-axis. (b) Side lengths are given on the *x*-axis and the sum of the ischemic area and burn area is given on the *y*-axis. (c) Replicate outcomes for thinned band pattern photocoagulation therapies at a constant length of 300 microns with normalized band areas corresponding to the different band widths on the *x*-axis and normalized ischemic area on the *y*-axis. (d) Replicate simulations were executed for band patterns with the widths given on the *x*-axis and the sum of the ischemic area and burn area given on the *y*-axis. (e) Replicate outcomes for shortened band patterns at fixed band width of 60 microns with normalized band areas corresponding to the different band lengths on the *x*-axis and normalized ischemic area on the *y*-axis. (f) Replicate simulations for band patterns with the lengths given on the *x*-axis and the sum of the ischemic area and burn area given on the *y*-axis.

We adapted the* dot pattern* and* band pattern* to achieve the optimal sum of burned area and ischemic area. For* dot pattern* therapy, the effect of decreased edge size from 100 microns to 80, 60, 40, and 20 microns was examined.* Dot pattern* therapy with *S* = 80 microns and *S* = 60 microns was shown in Figures 4(a) and 4(b), respectively. *S* = 80 case showed better effectiveness in preventing progression of ischemia than *S* = 60 case. In fact, *S* = 60 seemed a transition point in efficacy, as* dot patterns* with smaller sizes showed larger areas of ischemia (Figure 5(a)). Note that *S* = 20 microns' case reflected a theoretical minimum simulated burn size in the current model based on retinal cell size and showed little difference from control simulation. Although not all specific cases are shown in Figure 4, in the evaluation diagram, Figure 5, all cases were included. As for* band pattern* therapy, we considered two types of modifications: decrease in width and decrease in length. For the width-related type,* band pattern* burns were thinned with the intention of investigating a theoretical minimal size that could largely stop progression of diabetic capillary occlusion. The label used for this type is “*W*” (width). The widths simulated were 80, 60, 40, and 20 microns. For the length-related modification,* band pattern* burns were shortened equally from both ends to only cover the middle region of an A/V sector, for which the rationale was that retinal areas near the venule and arteriole are relatively well-oxygenated and protected against hypoxia. Lengths simulated were 50, 74, 100, and 124 microns. Two of the specific* band pattern* therapies are shown in Figures 4(c) and 4(d). Both cases effectively restricted progression of ischemia by creating barriers to propagation as shown in Figures 5(c) and 5(e) of the replicate simulations.

We evaluated these* dot and band patterns* with replicative simulations (Figure 5). In Figure 5, we first examined the effects on the replicative simulations for dot patterns of varying the burn size given as the side length of the square burn. Figure 5(a) shows the box and whisker plots of the predicted ischemic areas for the different total burn areas of the different dot sizes. More burn area gives less progression of ischemic area, as expected. In Figure 5(b) the summed area of ischemia and burns is given on the *y*-axis as a function of burn size. Here again the effects of increasing burn size and decreasing ischemic progression are largely complementary but over this size range are always well below the results with standard PRP. Also, similar to earlier results, the variability of ischemic progression drops at the larger burn size shown by the diminished height of the box. The effects of the variation in band width are given in Figures 5(c) and 5(d) and those for variation in band length are given in Figures 5(e) and 5(f). For these band cases there is no benefit to the increase in burn dimension. When widths of laser burns decreased from 100 microns (default in basic band pattern) to as small as 20 microns (roughly one cell size), the average ultimate ischemic areas were more or less unchanged, suggesting almost equally preventive power for different sizes. Similarly this holds for the length of these burns placed centered between arteriole and vein. This is therapeutically attractive, because during the optimization process total burned area was decreased almost 80%. Again, this is a theoretical model and there are considerable practical obstacles to the theoretically optimal placement of these small burns in the oxygen trough between the arterioles and venules.

The modelling conclusions are that dot pattern and band patterns of photocoagulation effectively prevented progression of diabetic capillary occlusion, as compared with traditional PRP treatment. Optimization of dot pattern therapy by shrinking the size of laser burns showed a critical size of gap between laser burns equal to about 140 microns, and for distances between laser burns greater than 140 microns increased areas of ischemia are seen caused by increased propagation of capillary occlusion (Figure 5(a)). This corresponds to burn sizes between 60 and 80 microns at *N* = 4 spacing. Optimization of band pattern therapies via either thinning or shortening the laser burn, on the other hand, displayed surprisingly consistent performance in prevention of ischemic propagation with small areas of ablation (Figures 5(c) and 5(e)). Out of all optimized burn patterns, *L* = 52 *μ* band pattern showed the smallest sum of retinal photocoagulation and ischemic area. This is consistent with the 140 *μ* gap for the dot pattern as this band length gives gaps of about 125 *μ* between the central band of laser treatment and the adjacent arteriole and venule. Note that the difference between dots and bands is superficially in terms of shape but that in this modelling dots are located adjacent to the arterioles and venules whereas bands are centered between the two vessels and this distinction is important with regard to the physiology of ischemic propagation.

**Figure 6:**
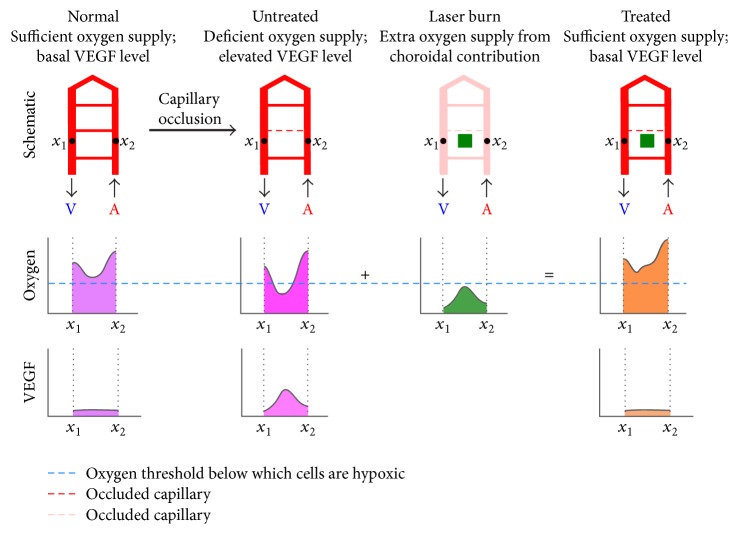
Oxygen and VEGF landscape shift following photocoagulation. Oxygen landscape shift after photocoagulation. The oxygen landscape exhibits peak tension near the arteriole and venule and trough tension near the center between these major vessels (left). Prior to capillary occlusion cells situated at the trough of oxygen tension are only slightly above the hypoxic threshold. Following capillary occlusion, the next subfigure shows the cells now fall below the hypoxia threshold and locally secrete VEGF and other factors propagating nearby capillary occlusion. Photocoagulation therapy, namely, the shortened* band pattern* therapy shown in the next subfigure as a green block, produces an extra oxygen peak at the center between the arteriole and venule. The additive oxygen tension from the choroid then relieves hypoxia, preventing elevation of VEGF synthesis and protecting against propagation of retinal capillary occlusion.

We demonstrate the presumed mechanism of suppression of ischemic propagation in a schematic of the oxygen landscape shift with laser treatment (Figure 6). In the normal state oxygen tension is high near the arteriole and venule and lower at the central region between vessels but no retina is hypoxic and therefor VEGF synthesis is at basal levels. This normal trough of oxygen tension is only slightly higher than the hypoxia threshold. Thus, capillary occlusion causes cells in these regions to become hypoxic with resultant increase in VEGF synthesis and resulting elevation of probability of adjacent capillary occlusion. The oxygen landscape shifts upward when the laser burn introduces an oxygen source between the arteriole and venule raising the oxygen level in the oxygen trough above the hypoxic threshold, preventing elevated synthesis of VEGF, and therefore preventing propagation of capillary occlusion.

## 4. Discussion

Peripheral retinal photocoagulation has a time honored validated importance in the treatment of diabetic neovascularization. We do not question this treatment for this purpose but ask whether a modified approach to peripheral photocoagulation can act to prevent the propagation of retinal capillary loss. Capillary loss produces retinal ischemia/hypoxia which causes synthesis of vascular endothelial growth factor which ultimately drives the propagation of neovascularization and some cases of macular edema [14, 15, 19, 27, 28]. Macular edema is the most frequent cause of moderate visual impairment in diabetics and neovascularization is the cause of severe visual loss [29]. Thus, prevention of progressive retinal ischemia could aid in preventing these major sight threatening complications. The model we have developed of locally propagating retinal ischemia due to an adverse positive feedback cycle [8] provides a framework within which to examine potential patterns of laser treatment and suggests alternative patterns of treatment which, within the model, seem to have advantages in the prevention of progressive capillary occlusion in diabetic retinopathy. We note that there is no actual validation of this approach to possible therapy but that this model reproduces many phenomena seen clinically in diabetic retinopathy [8] and does force some biophysically based thinking on a topic that has largely been dormant.

This paper's goal is to determine the best photocoagulation treatment with regard to preservation of visual function. Both peripheral laser photocoagulation and the consequences of the disease, retinal ischemia, yield a compromise in retinal function. In the DRS 11% of patients had a significant decline in visual acuity and 5% had a constriction of their visual fields [3]. Even threshold laser burns can cause visual field damage [30, 31]. Full-scatter PRP with argon laser significantly depressed peripheral visual field sensitivity though mild scatter improved peripheral visual field sensitivity [32] possibly showing the benefit of improved retinal oxygenation versus the damage from photocoagulation. Similar results on benefit to visual fields were obtained by Muqit et al. 2010 [33]. On the other hand, diabetic retinal ischemia itself also compromises visual functioning. Diabetics are visually compromised in several ways and certainly can show visual field defects prior to any laser treatment [34]. Visual fields are highly correlated with ETDRS Classification [28] of severity of retinopathy. Flicker ERG [35], color vision [36, 37], and contrast sensitivity [37] are all functionally impaired in diabetic retinopathy likely due to retinal ischemia. For these visual characteristics what we know is only the statement about the properties of diabetics' vision in general but we lack visual function parameters, such as visual thresholds, flicker fusion frequency, or color vision for local ischemic versus nonischemic areas of the diabetic peripheral retina except for the data of Chee and Flanagan (1993) [38]. Chee and Flanagan (1993) showed a strong correlation of areas of capillary nonperfusion with reduced retinal sensitivity on visual fields. To evaluate optimal treatment within the model we required a measure of total retinal compromise and decided, in the absence of more specific data on the level of visual function compromise in ischemic retina, to assume that a normal retinal photocoagulation produced the same functional deficit as retinal ischemia and therefore to sum total photocoagulated area and total area of retinal ischemia as the criterion to be optimized in the model treatment. We then tracked this total area as a function of time in the model with the ischemic areas measured over replicate computational simulations and the median as well as 25th and 75th percentiles given in the figures (because of the stochastic nature of the model, extent of ischemic areas varies from simulation to simulation within a replicate data set).

The model explored dot patterns and band patterns and varied relevant spatial parameters for each of these patterns within the peripheral capillary network modelled on peripheral retinal histopathology [25]. The standard PRP approach of “random” spacing of 250–500 microns' burns creates spaces between the burns of the same order of magnitude. This spacing is sufficient to allow propagation of capillary occlusion between the burns as these spaces are similar in size to the entire area we modelled. Because of this, spot sizes and spaces, as large as those used in current PRP treatment, were not separately modelled. We used 0.28 fraction of the retina photocoagulated based on simple calculations from the DRS study and assumed that the retina between the laser burns would have ischemic progression as in the untreated situation. The continued propagation of ischemia in retinal areas between the PRP laser burns may, by gradually increasing hypoxic retina and synthesis of VEGF, be a contributing factor to those patients who do not have regression of neovascularization after PRP or who frequently progress despite PRP. An alternative to standard PRP is targeted PRP which uses wide field angiography to detect the ischemic retinal areas and to only photocoagulate those areas, avoiding photocoagulation damage to nonischemic retina. This seems to be an effective approach to treatment of neovascularization [6, 7] but there are limited numbers of patients being treated this way and there is still no long term follow-up. The treatment philosophy in targeted PRP is that the VEGF causing the neovascularization is coming from the nonperfused retina so that only ischemic retina should be treated and any treatment of still perfused retina causes unnecessary retinal damage. There is no intent in targeted PRP treatment to address the issue of propagation of retinal ischemia dealt with in this paper.

We focus on a theoretical alternative of small closely spaced laser burns to retinal areas prior to the development of ischemia due to capillary occlusion. The proposed burns are considerably smaller than those utilized in standard panretinal photocoagulation and are preventative or prophylactic in that they are intended to prevent the development of retinal ischemia rather than to directly treat its ultimate adverse visual complications such as neovascularization. In the model they are placed with regard to the structure of the retinal vessels. Essentially all of the patterns modelled were superior to standard PRP in the prevention of ischemic propagation and result in a sum of ischemic and photocoagulated retina that is much smaller than that produced by current PRP treatment and therefore presumably results in less total visual compromise. For the most part, this reduction in ischemic propagation was not critically dependent on specific burn parameters. For example, in both Figures 5(c) and 5(e) the ischemic propagation showed no apparent dependence on width or length of the band within the range modelled. There were some differences however and the narrow band placed at the oxygen trough between the supplying arteriole and venule was the most effective in terms of the sum of photocoagulated and ischemic retina. The model predicts that this is what optimal photocoagulation treatment of nonischemic retina should be.

How would one validate this model treatment in actual diabetic retinopathy? The current selective PRP approach which treats only ischemic peripheral retina as determined by wide field angiography would seem an ideal starting point. The patients treated have areas of ischemic retina already lacking capillaries which are treated by standard PRP. Remaining nonischemic retinal areas with intact capillaries are left untreated though these same areas would be photocoagulated by those physicians who use the standard PRP approach. Certain ones of these nonischemic retinal areas could be treated using the modelled approach, either treating certain areas and leaving other areas untreated within a single eye or treating roughly half of each single nonischemic retinal area while leaving the other half untreated. Follow-up with wide field angiography quantifying the areal changes in ischemia should allow ready determination of the effectiveness of this potential approach. Since the treatment is within a single eye of a single patient, this approach would avoid the many issues of variability of disease progression across patients seen in diabetic retinopathy and thereby reduce the size of a clinical study needed for verification. Also this would lie between two clinical approaches already utilized which either photocoagulate these areas, the standard PRP approach, or do not treat them at all, the selective PRP approach. This would seem to allow a low hurdle for this sort of clinical study. Ideally this work would be done in a primate before being tested in humans but under current conditions limiting primate availability for experimentation, which would be much more difficult to accomplish but the approach would be the same. The actual patterns of retinopathy seen in other animals, such as rodents, do not duplicate the full pattern of retinopathy seen in humans, for example, their absence of neovascularization, and would therefore not likely be useful as models for treatment.

The obvious limitation of this work is that it is a modelling study in which the physiological model of the propagation of retinal capillary occlusion is based on data from a number of species determined under a range of experimental situations from cell cultures to* in vivo* primate retinal angiography and histopathology. Very little of this support for the mechanism of capillary occlusion in diabetes has been obtained in humans. We are assuming in the model that these various physiological mechanisms are applicable to the human diabetic retina. All factors which may be important in the propagation of capillary occlusion are not able to be modelled and many details are treated as “black boxes.”

In summary, a physiologically based computational model of the progression of diabetic retinal ischemia via a local adverse feedback cycle of capillary occlusion produces predictions about possible preventative retinal laser photocoagulation acting to preserve nonischemic retina by preventing propagation of local retinal capillary occlusion. This form of laser treatment would be quite distinct from conventional retinal photocoagulation, PRP, as it is currently utilized to treat diabetic neovascularization largely in that it would utilize much smaller burns of greater number and is intended to preventatively treat still vascularized nonischemic retina. Prevention of progression of diabetic retinal ischemia could be of major benefit in the early treatment and prevention of the visual complications of diabetic retinopathy.
